# IvCDS: An End-to-End Driver Simulator for Personal In-Vehicle Conversational Assistant

**DOI:** 10.3390/ijerph192315493

**Published:** 2022-11-22

**Authors:** Tianbo Ji, Xuanhua Yin, Peng Cheng, Liting Zhou, Siyou Liu, Wei Bao, Chenyang Lyu

**Affiliations:** 1 School of Transportation and Civil Engineering, Nantong University, Nantong 226000, China; 2 School of Informatics, University of Edinburgh, Edinburgh EH8 9YL, UK; 3 Alibaba Group, Hangzhou 311121, China; 4 ADAPT Centre, School of Computing, Dublin City University, D09 DXA0 Dublin, Ireland; 5 Faculty of Languages and Translation, Macao Polytechnic University, Macao, China; 6 China Electronics Standardization Institute, Beijing 101102, China; 7 SFI Centre for Research Training in Machine Learning, School of Computing, Dublin City University, D09 DXA0 Dublin, Ireland

**Keywords:** transportation and interdisciplinary application, driver–vehicle interaction, machine learning, natural language processing, task-oriented dialogue

## Abstract

An advanced driver simulator methodology facilitates a well-connected interaction between the environment and drivers. Multiple traffic information environment language processing aims to help drivers accommodate travel demand: safety prewarning, destination navigation, hotel/restaurant reservation, and so on. Task-oriented dialogue systems generally aim to assist human users in achieving these specific goals by a conversation in the form of natural language. The development of current neural network based dialogue systems relies on relevant datasets, such as KVRET. These datasets are generally used for training and evaluating a dialogue agent (e.g., an in-vehicle assistant). Therefore, a simulator for the human user side is necessarily required for assessing an agent system if no real person is involved. We propose a new end-to-end simulator to operate as a human driver that is capable of understanding and responding to assistant utterances. This proposed driver simulator enables one to interact with an in-vehicle assistant like a real person, and the diversity of conversations can be simply controlled by changing the assigned driver profile. Results of our experiment demonstrate that this proposed simulator achieves the best performance on all tasks compared with other models.

## 1. Introduction

Transportation-related issues are highly related to the road/driver safety and traffic environment, and are attracting growing interests [1,2,3,4]. For example, fatigue driving can significantly raise the possibility of car accidents [5], and is generally influenced by driver-related factors such as the state of sleep and health [6]. The developing intelligent driving techniques are deemed to be capable of providing feasible approaches to addressing such issues [7,8], and recent research shows a propensity of utilizing intelligent vehicle systems (e.g., a driver assistant system or a driver behavior-recognition system). These systems are designed to detect the real intents behind human driver behaviors and adopt relevant measures [9,10], resulting in the improvement of driving safety and efficiency. Despite the prevalence of the application of intelligent driving however, it additionally raises the concern that these newly advanced techniques may even lead to traffic accidents due to fatal errors such as causing driver distraction or taking wrong actions [11].

Meanwhile, because current work mainly focuses on the system side, a driving simulator is an appropriate technique which provides approaches to simulating how real drivers interact with the driving environment (e.g., an intelligent driving system). There exists practical applications of driving simulators which involve a wide range of traffic areas and vehicle techniques, such as advanced driver assistance systems [12], driver education in driving automation [13], assessment of safety at signalized intersection [14], and so on. For example, Simović et al. [15] employ an in-laboratory driving simulator to investigate how different factors can influence the e-bicycle speed perception of human drivers.

Hence, we propose a conversational driver simulator in this paper by leveraging the task of dialogue systems, which can potentially facilitate driver safety by increasing the quality of drivers’ interaction with in-vehicle assistant systems. Generally speaking, dialogue systems aim to generate appropriate responses to user input, and it is an important research direction not only due to its practical applications but also for its direct connection to artificial intelligence (AI) [16,17]. Dialogue systems can be categorized into two classes according to their applications: (1) open-domain dialogue systems that aim at synthesizing human-like conversations with users [18,19,20], and (2) task-oriented dialogue (TOD) systems of which the goal is to help human users to complete certain tasks such as virtual assistant [21,22,23].

In the two main classes of dialogue system, task-oriented dialogue system has been the focus of the research community as it has many valuable applications such as an online booking system, counselling service, and in-vehicle assistant [22,24]. In recent years, significant progress has been made in the development of task-oriented dialogue systems beginning with the deployment of neural models, especially pretrained language models (PLM) [25]. Task-oriented dialogue systems based on neural networks (especially PLMs-based models) trained on relevant dataset have shown superior performance on capturing user’s intent and generating proper response [26,27,28,29]. However, such success heavily relies on the availability of relevant datasets such as MultiWOZ, KVRET and BANKING77 [24,30,31], which usually needs rich human resources to construct because the annotation of user intent and response requires interaction with human annotators. Therefore, such a dataset is expensive to obtain, especially when the amount of required data is large, as neural models typically need more training data to achieve competitive performance. There is no doubt that the large amount of resource needed to build such datasets has undesirably hindered the development of task-oriented systems. To address this problem, we propose to adopt the idea of using a simulator as a human user whose potential applications include evaluating a task-oriented dialogue system or augmenting data to alleviate the shortage of datasets [32,33]. The primary purpose of user simulator is to have a dialogue model acting as the human user, which then can be used to converse with the task-dialogue system and thus automate the evaluation of such systems without requiring human evaluators [34,35,36].

Current work of simulators in the user side generally involves agenda-based simulation, which entirely relies on handcrafted rules [32,37]. Such methods are deemed too costly to be practical and suffer from a lack of generalization. In addition, some research proposes end-to-end user simulators [35,38] as an approach to conducting reinforcement learning, and these simulators are expected to serve TOD systems rather than act as practical standalone simulators.

In this paper, we propose in-vehicle conversational driver simulator (IvCDS), an end-to-end PLM-based simulator that can play the role of human drivers during the interaction with an assistant in the task-oriented dialogue task. First, we process KVRET, a TOD dataset consisting of conversations between a human driver and an in-car assistant, to obtain an appropriate dataset for the training and inference of a driver simulator. The contents in KVRET are re-labeled by using existing information, and the original driver-assistant conversations are converted into the assistant-driver format. Then, we train IvCDS with the training objective of language modelling on processed KVRET. Finally, we investigate the performance of IvCDS on three distinct tasks: NLU, POL, and NLG, which will be introduced in Section 2 on the test set of KVRET. We demonstrate that the proposed IvCDS can outperform existing state-of-the-art (SOTA) models on aforementioned tasks. Furthermore, we conduct ablation studies to investigate the importance of different components of IvCDS.

### Paper Structure

To provide a clear picture of this paper, we will briefly introduce the overall paper structure in this section. Section 2 reviews the background and related research of the TOD task, as well as its three subtasks. In addition, Section 3 introduces the approach to processing the dataset we used, and gives the detailed methodology of our driver simulator. Next, Section 4 compares the performances of our driver simulator and other PLM-based baselines, and reports the results of the ablation study. Finally, Section 5 depicts the summary of this paper, and provides the discussion about potential research directions and practical applications in the future.

## 2. Related Work: Task-Oriented Dialogue Systems

In general, a task-oriented dialogue system [21,22,23,39,40,41] adopts one of the following architectures: a conventional pipeline system or an end-to-end neural model [42]. The pipeline scheme is a modular architecture consisting of four components: natural language understanding (NLU), dialog state tracker (DST), dialog policy (POL), and natural language generation (NLG). Traditionally, models for different components are separately trained and evaluated on task-specific datasets [43]. For example, BANKING77 provides a dataset composed of paired utterances and intents which is suitable for NLU and NLG tasks [31]. Meanwhile, a recent trend is to jointly train pipeline modules in an end-to-end manner instead of combining models that are separately trained. Note that DST is not involved in a user simulator [44], because the user policy is directly generated according to given assistant actions without the belief state.

### 2.1. Natural Language Understanding

Natural language understanding (NLU) aims to understand the intents and actions of an utterance. It can portray conversational actions as a set of intents and slot values. Intents are expressions of the reason why the speaker issued the sentence, such as queries and notifications, and are the utterance’s slot-values are specific to the task and content mentioned in the utterance. Based on the structure of conversational behaviour, NLU can be divided into two parts, intent detection and slot-value extraction. Its tasks are based on a series of tokens as input to the model, and RNNs and their variants are powerful for handling such sequence modelling and are widely used for intent detection and slot-value extraction [45]. In addition, the recent pretrained model BERT is another popular choice [46]. In case of a driver simulator, NLU focuses on understanding the intents behind the speaking of an assistant.

### 2.2. Dialog Policy

For a user–agent dialogue, the input to dialog policy (POL) is the current conversation state consisting of slot-value pairs representing the user’s intents [42,47,48,49], and it generates the system actions. A typical approach is to train a dialog policy based on a conversation corpus by using supervised or simulated learning, and then fine tune the model by using reinforcement learning [50,51,52,53], because it would be too costly to be practical if human users (e.g., experts or volunteers) are involved. In terms of a driver simulator, POL aims to generate actions of a driver according to assistant actions.

### 2.3. Natural Language Generation

Natural language generation (NLG) maps POL-generated dialogue acts to textual sentences, and is often modelled as a conditional language generation task [54,55,56]. It receives a set of behaviours as input, and generates a textual response as output in the form of meaningful and fluent natural language. The generated response is expected to properly understand the user intents and to give appropriate actions from the user perspective. For a driver simulator, NLG operates as a human driver in response to an assistant with appropriate textual utterance.

## 3. Methodology

In this section, we introduce how we convert the KVRET dataset into the appropriate shape, including data cleaning, labelling and formatting. We additionally provide the methodology of the driver simulator.

### 3.1. Data

KVRET is a task-oriented dialogue dataset consisting of the conversations between a driver and an in-vehicle assistant [24]. It is a multidomain multiturn dataset which involves three scenarios: calendar scheduling, weather information retrieval, and point-of-interest (POI) navigation. These distinct scenarios aim to enhance the diversity of driver-assistant conversations for promoting the development of an experienced and comprehensive personal vehicular assistant. Similar to other task-oriented dialogue datasets such as MultiWOZ [30] and RiSAWOZ [57], KVRET likewise utilizes the Wizard-of-Oz scheme [58] as the approach of collecting conversations and labels. However, because the original dataset mainly focuses on the assistant side, some labels that are essential for a driver simulator (e.g., driver actions) are unfortunately missing. We need to extract the actions of the driver and assistant in each conversation as an important feature to train the user simulator.

#### 3.1.1. Assistant Actions

A driver simulator is expected to have the ability of understanding the intent and actions behind the utterance from the assistant. Assistant actions are indispensable to a driver simulator for both NLU and POL tasks.

**Figure 1:**
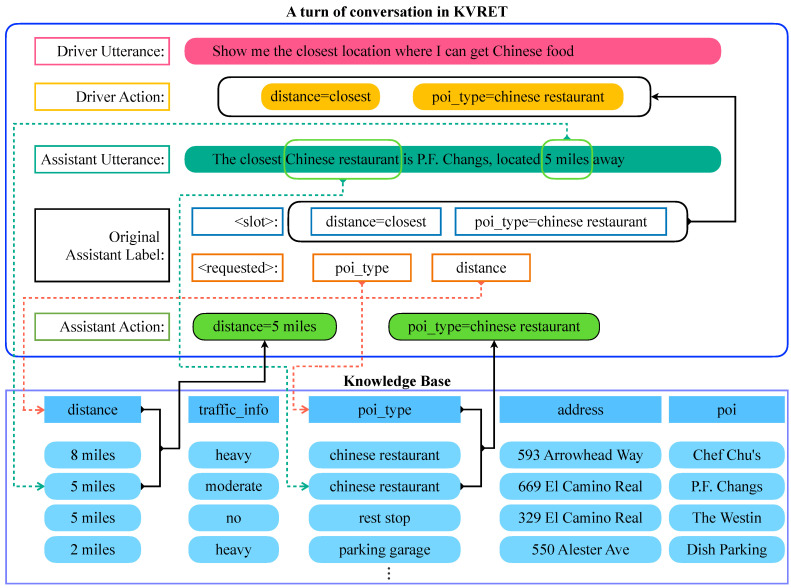
The overview process of labeling the driver/assistant actions using the given utterances and original labels in the KVRET dataset.

Despite the unavailability of such labels in the original KVRET dataset, we leverage the provided information to label the actions of assistant utterances in the correct form we need. For each conversation, KVRET gives a knowledge base containing a set of key-value pairs which describe all possible actions that an assistant can take. In addition, each assistant utterance in a conversation is accompanied by the <requested> label, which contains all keys that can be used to locate values in the knowledge base. When investigating the the original dataset, we found that the types and manners of actions can vary according to different scenarios, resulting in different extraction strategies. For calendar scheduling and POI navigation, the <requested> label generally involves location, time and events. In this case, the corresponding actions simply depends on the matched items between the knowledge base and the the <requested> label. Figure 1 gives an example of labeling assistant actions on the POI navigation scenario. By matching the keys (i.e., *poi_type* and *distance* in <requested>) and values (i.e., *5 miles* and *chinese restaurant* in the assistant utterance) in the knowledge base, we can therefore determine that the assistant action should be *[distance] = 5 miles* and *[poi_type] = chinese restaurant*.

For scenarios regarding weather information retrieval however, the direct matching strategy is inappropriate because the knowledge base has a different format. The keys in it are the day of a week (i.e., Monday to Sunday), and the corresponding value of each key contains weather attributes and the minimum and maximum temperatures. For slots that additionally contain the date information, the date will be used to match the key in the knowledge base to determine the actions. Otherwise, each group of temperature information will be divided into three parts: weather attribute, lowest temperature, and highest temperature. The detailed matching process is illustrated in Algorithm 1, where KB is the knowledge base.
**Algorithm 1:** The processing step of extracting assistant actions for the weather information retrieval scenario.S← a set of key-value pairs in <slots> ▹ weather_attribute = blizzard, date = Friday, ⋯$S_{keys}\leftarrow$ all keys in *S*S[key]← the value of key in *S*$K_{i}\leftarrow$ the *i*-th item in KB$K_{keys}\leftarrow$ all keys in KB                   ▹ monday, tuesday, location,⋯$K_{i}\left[key\right]\leftarrow$ the value of key in *i*-th item in KB     ▹ "snow, low of 60F, high of 80F", ⋯a← the answer of assistant      ▹ On Friday it’s gonna rain in Brentwood, ⋯actions← the actions of assistant**if** $location\notin S_{keys}$ **then**    actions← a question about location**else**    get $K_{i}$ where S[key] in $K_{i}$    **if** $date\in S_{keys}$ **then**        actions←location = $K_{i}\left[location\right],date$ = $K_{i}\left[date\right]$    **else**        split $K_{i}\left[key\right]$ into three parts        **if** anyparts∈a **then**           actions←location = $K_{i}\left[location\right],key$ = $K_{i}\left[key\right]$        **end if**    **end if****end if**

#### 3.1.2. Driver Actions

Driver actions indicate the actual objectives beneath the driver utterance. In this experiment, driver actions are necessary for the POL and NLG task. Although no label is available on the driver side because KVRET mainly focuses on the assistant side, the driver actions can be found in the <slot> from the assistant labels which is provided for the NLU task in the original dataset. We take the content of <slot> in each assistant response as the actions of its previous driver utterance. For example in Figure 1, the action of driver utterance “Show me the closest location where I can get Chinese food” are *[distance] = closet* and *[poi_type] = chinese restaurant* from the original <slot> label of its subsequent assistant response. We additionally add greeting labels, such as *[greeting] = thank* for driver utterance “Thank you”.

#### 3.1.3. Driver Profile

For the driver simulator, we use the driver profile to indicate the overall objective of the driver’s interaction with the in-vehicle assistant, including all intents and actions of the driver throughout the entire conversation. For the training and inference in our experiment, each driver profile is comprised of all driver actions in a conversation. The driver profile performs as a pool of potential actions which can help to determine the next actions on the POL task. Meanwhile, for practical application in the future, a driver profile can be generated by extracting potential actions from the knowledge base. By changing the driver profile, a driver simulator is expected to have different behaviours.

#### 3.1.4. Reordering

**Figure 2:**
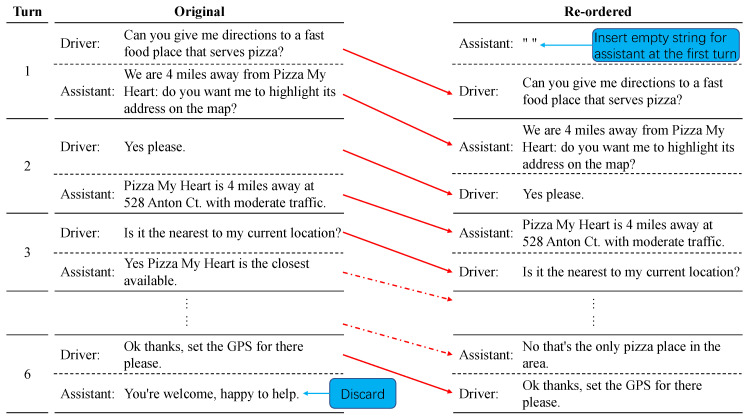
An example where an original conversation is converted into the assistant-driver format.

The original dataset consists of driver–assistant conversation, meaning that a conversation is always started by the driver. We convert conversations into an assistant–driver format as we expect each turn of a conversation can have an input utterance from the assistant to guarantee the exact NLU-POL-NLG structure. Specifically, the driver data at *i*th turn together with the assistant data at (i−1)th turn composes the new *i*th assistant-driver turn in the processed dataset. In particular, the driver data at the first turn will be accompanied by the assistant data whose utterance and actions are empty, to constitute the new first turn. Additionally, the assistant data at the last turn will be discarded as no corresponding driver data is available. Figure 2 provides an example.

### 3.2. Model

**Figure 3:**
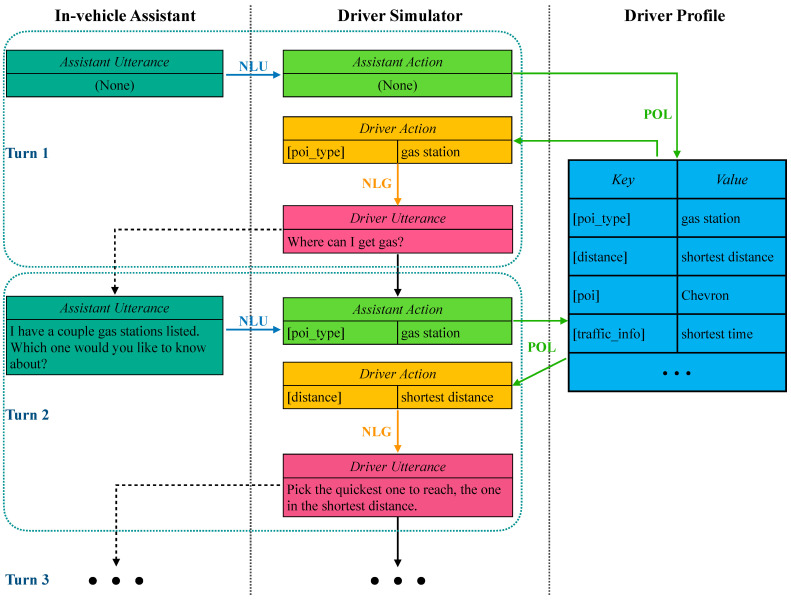
The overall structure of how the driver simulator interact with an in-vehicle assistant.

Figure 3 illustrates the overall process of the driver simulator when conversing with an in-vehicle assistant, with regards to three tasks: NLU, POL, and NLG. In general, the driver simulator first receives an utterance from the assistant, and the assistant action, the driver action and driver utterance are sequentially generated. We will then introduce the means of the driver simulator to handle these tasks in detail.

#### 3.2.1. NLU

The NLU task expects the driver simulator to produce the actions according to a given assistant utterance. Given an assistant utterance $AU_{t}$ at turn *t* together with the driver profile DP and the dialogue history $H_{t}$, Equation (1) describes how the simulator generates the corresponding actions on the NLU task as follows:

(1) $$\begin{array}{cc} AA_{t} & ={\mathrm{NLU}}_{M}\left(\left[DP;H_{t};AU_{t}\right]\right), \end{array}$$

where $AA_{t}$ means the generated assistant actions, *M* represents the model of driver simulator, and the dialogue history $H_{t}=\left[C_{1};C_{2};\cdots ;C_{t-1}\right]$ is made up of the conversation contents $C_{i}$ of all previous turns. The conversation content $C_{i}$ at turn *i* is composed of the concatenation $C_{i}=\left[AU_{i};AA_{i};DA_{i};DU_{i}\right]$ where AU = assistant utterance, AA = assistant action, DA = driver action $DA_{i}$ and DU = driver utterance.

For example in Figure 3 at turn 2, the assistant says “I have a couple gas stations listed. Which one would you like to know about?”, and the NLU task expects the simulator to generate the action as *[poi_type] = gas_station*.

#### 3.2.2. Pol

After confirming the actions behind an assistant utterance on the NLU task, POL then requires the driver simulator to take corresponding actions. At turn *t*, the driver action (DA) is generated as described in Equation (2):

(2) $$\begin{array}{cc} DA_{t} & ={\mathrm{POL}}_{M}\left(\left[DP;H_{t};AU_{t};AA_{t}\right]\right), \end{array}$$

where $AA_{t}$ is the output of Equation (1).

As shown in Figure 3, the driver profile provides all potential actions that a driver can take, and the simulator then generates the action *[distance] = shortest distance* in response to the assistant action that is generated from previous NLU task after referring to the given driver profile.

#### 3.2.3. Nlg

NLG is the task which translates the generated action into the utterance in the form of natural language. The driver simulator *M* will produce the driver utterance at turn *t* according to Equation (3), combining with the outputs from previous two tasks:

(3) $$\begin{array}{cc} DU_{t} & ={\mathrm{NLG}}_{M}\left(\left[DP;H_{t};AU_{t};AA_{t};DA_{t}\right]\right), \end{array}$$

where $AA_{t}$ and $DA_{t}$ are the output of Equation (1) and Equation (2), respectively.

For example, the simulator is capable of converting the corresponding driver action *[distance] = shortest distance* into the textual sentence “Pick the quickest one to reach, the one in the shortest distance”, as shown in Figure 3.

#### 3.2.4. Training and Inference

IvCDS is based on GPT-2 [59], a unidirectional language model which consists of a transformer decoder and has been pre-trained on large-scale datasets, therefore training IvCDS is actually fine tuning the pretrained GPT-2. Meanwhile, the training objective of IvCDS is the objective of a language model, needing no additional objectives such as next sequence prediction. Given a training sequence $S=\{w_{1},w_{2},w_{3},\cdots ,w_{n}\}$, it is expected to learn next word prediction by maximizing the likelihood of the next token as Equation (4):

(4) $$\begin{array}{cc} L & =\sum_{i=1}^{n}logP(w_{i}|\left\{w_{1},w_{2},\cdots ,w_{i-1}\right\}) \end{array}$$

where $w_{i}$ is the *i*-th word/token in the training sequence *S*, and *P* is the probability of a token given all its previous tokens.

**Figure 4:**
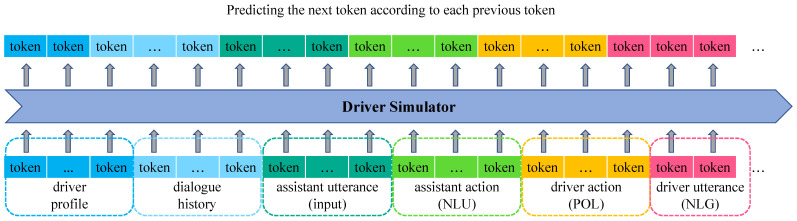
The process of training the simulator which learns to generate the next token according to previous tokens.

Figure 4 illustrates the training of IvCDS. In addition, it requires IvCDS to have the ability of stopping token prediction at a proper time for each task. Hence, we wrap the constituents in the input sequence with different special tokens to indicate the category of a constituent and to instruct IvCDS to distinguish the current task. Specifically, five categories of constituents are used, as shown in Figure 4 with different colors: driver profile, assistant utterance, assistant action, driver action, and driver utterance. The beginning and ending of a constituent are indicated by “[soxx]” (start of xx) and “[eoxx]” (end of xx), respectively. Special tokens for these constituents are introduced as follows:
driver profile:[sodp]and[eodp]assistant utterance:[soau]and[eoau]assistant action:[soaa]and[eoaa]driver action:[soda]and[eoda]driver utterance:[sodu]and[eodu].

**Figure 5:**
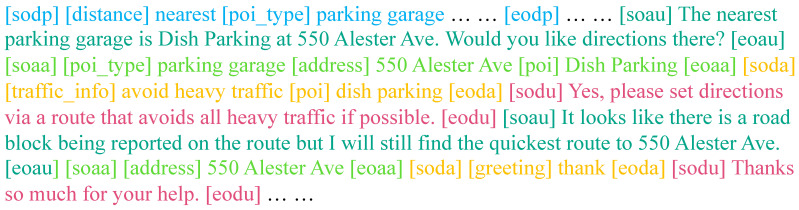
An example from the dataset where colored contents indicates different categories of constituents.

Note that, despite the six different colors in Figure 4, the dialogue history in fact consists of driver&assistant utterances and actions from previous turns, and we do not use any specific token for it. We therefore describe five categories of constituents. In addition, Figure 5 gives an example input sequence from the processed KVRET training set for the training of IvCDS.

**Figure 6:**
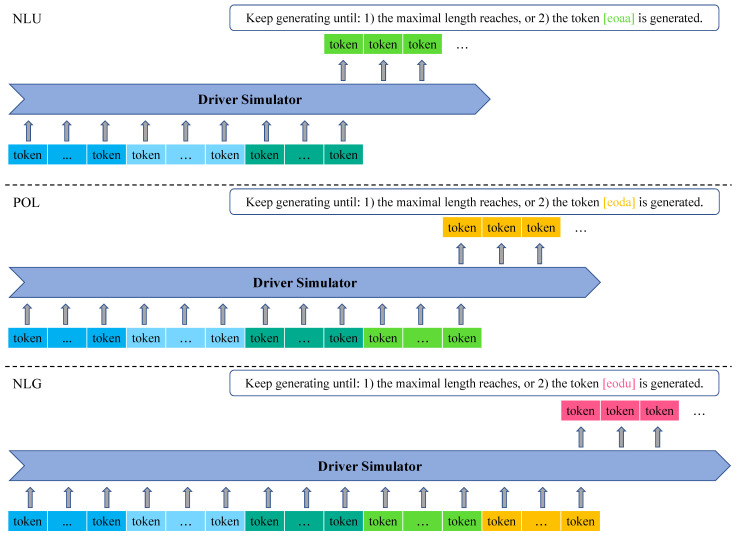
The process of inferring on the three tasks after the model training is completed.

When the training step is completed, we believe that IvCDS achieves the abilities of understanding utterances from the assistant and makes the decision of actions to take using the history of dialogue and pre-assigned driver profile. Subsequently, the trained IvCDS can be evaluated according to the results of its inference on the test set. Figure 6 shows the inference process on different tasks, where the input sequence for each task consists of necessary constituents and the driver simulator is expected to sequentially generating tokens until the termination condition is achieved. For example, once IvCDS outputs the special token “[eoda]”, the token prediction stops during the POL task. In addition, prediction will be terminated as well if the length of generated sequence reaches a predefined maximum, no matter whether the task-specific token is generated.

## 4. Experiment

To encourage reproduction of our work, we introduce how the experiment of IvCDS is carried out in detail in this section, including the involved baseline models, the hyperparameters of training IvCDS and baselines, and the evaluation methods for different tasks. In addition, we compare the performances of IvCDS and other baselines by reporting their results on three tasks. Furthermore, we conduct the ablation study to investigate the influence of dialogue history and driver profile on IvCDS.

### 4.1. Experiment Setup

In this experiment, the training and inference entirely depends on one NVIDIA GeForce RTX 3090 graphics card. For the training parameters of IvCDS, we set the learning rate as 5e-5, the batch size as 4, and the number of epochs as 40. The pretrained model and tokenizer follows the standard implement of GPT-2 model from HuggingFace’s Transformers [60] with 32 additional special tokens, including 10 separator tokens like “[sodp]” and “[eoau]”, and 22 action key tokens such as “[poi]” and “[poi_type]” (see Figure 5). In terms of inference, we limit the maximal length of candidate outputs as [lengthofinput]+80, and task-specific termination tokens are: “[eoaa]” for NLU, `[eoda]” for POL, and “[eodu]” for NLG.

#### 4.1.1. Baseline Models

To investigate the performance of IvCDS, we additionally include eight models as baselines for comparison such as BERT [61], BART [62], ProphetNet [63], PEGASUS [64], T5 [65], etc. Recently, these baselines have successfully achieved state-of-the-art performance on various NLP-related tasks [66,67,68,69,70] whose pretrained model weights are publicly available on HuggingFace’s Transformers [60] as well. Table 1 gives a brief review of these baselines.

The aforementioned baselines are pretrained models and are fine tuned on the processed KVRET training set for each task. The learning rate is likewise 5e-5, and each baseline is trained with at least 20 epochs to minimize the training and validation loss. The batch size varies according to both the parameter size of baselines and payload of the graphics card, ranging from 2 to 32. The action key tokens such as [poi] are added to these baselines as well for fair comparison.

#### 4.1.2. Evaluation Metrics

For NLU and POL tasks, since the outputs are actions in the form of key-value pairs, we use precision, recall, and f-measure for their evaluation. Items, namely key-value pairs, in the outputs for NLU and POL tasks are compared with the items in the references on test set at turn-level. Strict exact match is applied, resulting in penalization of redundancy items.

For the NLG task, the generated sentences are evaluated by word overlap-based evaluation metrics, which are commonly applied for assessing the quality of textual sequences in generative NLP tasks, such as question generation [75] and open-domain dialogue systems [20]. Four metrics as follows are employed:

- BLEU [76]: The bilingual evaluation understudy (BLEU) is an evaluation metric which assesses the quality of a generated candidate by the precision of the *n*-grams between it and its corresponding references. In this experiment, we computed the BLEU-4 score, which uses equally weighed 1∼4 grams.
- GLEU [77]: Google-BLEU (GLEU) is a variety of BLEU. Instead of the standalone precision, GLEU computes the precision and recall of *n*-gram between a candidate and a reference. The minimum between precision and recall is then uses as the GLEU score, and *n* is typically chosen as 4.
- ROUGE-L [78]: ROUGE-L is the widely applied variant of recall-oriented understudy for gisting evaluation (ROUGE), a recall-adapted version of BLEU, wherein L denotes to the longest common subsequence (LCS). It computes the precision and recall using the LCS between a candidate and a reference, rather than *n*-gram.
- METEOR [79]: Metric for evaluation of translation with explicit ordering (METEOR) is the evaluation metric that was initially proposed to remedy the known weaknesses of BLEU (e.g., BLEU does not consider recall and performs inaccurately when evaluating at the sentence level). In addition to exact match, METEOR uses other strategies such as synonyms mapping to match the uni-gram between a candidate and a reference, and the METEOR score is computed by the precision and recall.

To achieve a fair comparison, the following preprocessing strategies are applied on raw model outputs before their evaluation: (1) separator tokens such as [eoau] and [eodp], and punctuation are discarded; (2) model-specific special tokens are removed; (3) outputs are lower-cased.

### 4.2. System Performances

Table 2 reports the results of our proposed driver simulator and other baseline models on the three tasks: NLU, POL, and NLG, where these models are evaluated based on their outputs on the processed KVRET test set. In general, we find that IvCDS successfully outperform other models on all tasks according to most evaluation metrics.

First, IvCDS has the best performance on the NLU task, and the gap of F1 scores between it and the second-ranked model, namely ProphetNet, achieves 4. It denotes that IvCDS properly plays the role of a human driver that can felicitously realize the intent behind the utterances from an in-vehicle assistant. In addition, an interesting observation is that, most baselines can have a relatively high level of recall, despite the low precision and f1 scores. This may imply that, these models tend to generate a large number of predictions which are maximally capable of covering the items in references; nonetheless, most of the predicted items are incorrect. Such a situation is obvious in terms of BERT2BERT, as its recall reaches as high as 87 with an extremely low precision of less than 20.

With respect to the POL task, we find that IvCDS still achieves the highest precision and F1 score, but the recall is slightly lower than BART-large. The gap of F1 scores between IvCDS and the second-ranked Pegasus is more than 9, whereas the gap of recall between IvCDS and BART-large is only about 2. Similar to results in the NLU task, the high-recall low-precision situation appears again on these baseline models. We additionally find that, models, including IvCDS and most baselines, perform better on NLU than POL, whereas the probable reason may be the POL task requires the additional ability of making decision by retrieving useful information from the driver profile. Meanwhile, an opposite trend appears on Pegasus and BigBird, and such models probably do well in understanding structured information like the assistant actions and driver profile, but lack the ability of understanding text in natural language.

For NLG task, IvCDS is the best among all models according to BLEU-4, ROUGE-L and GLEU metrics but slightly worse than BART-large on METEOR scores. Because METEOR is the only metric that relies on uni-grams, which is generally a single token, we think this implies BART-large is prone to produce correct tokens; however, the token order may differ from the reference, resulting in lower scores of evaluation metrics which depend on *n*-grams (n>1) or LCS.

### 4.3. Ablation Experiment

Ablation study is proposed to investigate how important a certain part of a neural network is [80]. In our experiment, we concatenate driver profile and dialogue history, with the task-essential input components (e.g., assistant utterance for NLU) as the input sequence, and we would like to determine the degree to which these two components can have an influence on the performance of IvCDS. Table 3 reports the results of the ablation experiment, where training means whether a model is trained with specific components in training sequences, inference means whether the components are included in input sequences, and *H* and DP are dialogue history and driver profile, respectively.

#### 4.3.1. Ablated Training & Inference

The first three models fine tune GPT-2 by using the same training objective as described in Section 3.2.4, and the training sequences follow a structure similar to Figure 5 with different combinations of *H* and DP. The input sequences use the same combination as training when inferring on the test set. Compared with the original IvCDS (O-IvCDS), namely the last model in Table 3. We find that these IvCDS models with ablated training and inference (A-IvCDS${}_{T\&I}$) appear with varying degrees of performance reduction on different tasks.

For the NLU task, the F1 score of these models can still achieve at least 70, and we think this indicates that A-IvCDS${}_{T\&I}$ somewhat gains the ability of understanding the intents behind assistant utterances without DP and *H*. In addition, an interesting observation is that, the A-IvCDS${}_{T\&I}$ without *H* and DP performs better than those which contain either *H* or DP. We think the potential explanation could be that, the standalone use of one such component may import extra disturbance items during training, resulting in the model’s failure of utilizing implicit information in the component during inference.

Meanwhile, their POL performance drops to as low as around 30 in terms of F1 scores, meaning that these models failed to make the decision of a driver to respond to the assistant. Moreover, A-IvCDS${}_{T\&I}$ with DP can have a better performance than those without DP, which denotes that, the driver profile which is expected to operate as the knowledge base, can have a positive influence on the POL task

In terms of NLU, all models seem to perform faultily with an evident decrease of word overlap-based evaluation metric scores, compared to O-IvCDS, revealing that training without the joint use of *H* and DP will negatively affect models’ ability of producing meaningful and fluent sentences in natural language.

#### 4.3.2. Sole Ablated Inference

The fourth to sixth models in Table 3, called A-IvCDS${}_{I}$, represent the ablated IvCDS models which are in fact O-IvCDS but with ablated inference using varying combinations of *H* and DP.

For NLU and NLG, we can observe a decrease on metric scores but they are barely less than O-IvCDS, whereas A-IvCDS${}_{I}$ with DP even shows an increase on METEOR scores. Among them, the model with neither *H* nor DP shows the worst performance. Therefore, we think the information in these two components still help to detect assistant intents and generate meaningful responses during inference, although A-IvCDS${}_{I}$ has gained corresponding ability during training.

With respect to POL, despite the decrease of scores in general, there exist striking levels of difference of A-IvCDS${}_{I}$ with varying component combinations. Models without DP, regardless of the inclusion of *H*, perform badly at F1<30, whereas the one with DP achieves F1≈76. We think this precisely reveals the importance of driver profiles on the POL task, which additionally emphasizes our contribution of proposing the processed KVRET dataset because it can provide the necessary driver profile for a driver simulator.

## 5. Conclusions and Future Work

In this paper, we focus on proposing a simulator which can act as a human driver having the abilities of assistant intent perception, making decision and generating appropriate and fluent responses. Two main contributions we made in this paper are: (1) we processed the KVRET dataset into the appropriate format for a driver simulator; (2) we proposed IvCDS, a PLM-based driver simulator. We demonstrated that IvCDS successfully achieves the best performance on all three tasks—NLU, POL, and NLG—compared with existing SOTA models. We subsequently carried out an ablation experiment, the results of which show that the joint use of dialogue history and driver profile can positively affect the performance of IvCDS, whereas the latter plays an important role in improving the performance of a driver simulator on the POL task.

For research in the future, we would first like to further investigate the reason why models trained without any dialogue history or driver profile outperform others which contains one of them according the results of ablation study. Because we think this may imply potential noise in our processed dataset, we will focus on filtering out the undiscovered labeling errors in it by utilizing heuristic algorithms or by expert annotators. In addition, a potential direction of improving IvCDS is to increase its recall in POL since it was found to be slightly lower than BART-large. We think error analysis would be useful for achieving such a goal. Moreover, we are encouraged to examine or adapt this driver simulator on more relevant TOD datasets in the future.

In terms of practical applications of driver simulator, we would like to use IvCDS to interact with in-vehicle assistant systems for evaluation, and this can help to find their weaknesses according to the results of interaction. We believe the improvement of the performance of in-vehicle assistant systems can additionally promote the development of intelligent driving techniques. In addition to the importance of scientific research, the driver simulator is important for the education of all road users as well. A driver simulator can helping to solve transportation-based issues in terms of traffic environment and road safety, by practicing their abilities, skills, and knowledge about traffic in a safe environment, for themselves and other traffic participants.

## Figures and Tables

**Table 1:** Involved baseline models in this experiment and their details.

Model	Description
BART [62]	An approach which utilizes a transformer-based [71] denoising auto-regressive decoder combined with a transformer-based bidirectional encoder to pretrain a seq2seq model. In this experiment, BART-large and BART-base are examined.
BigBird [72]	A mechanism which uses sparse attention to enable transformer-based models to handle long sequences.
Blenderbot [73]	A transformer-based model trained on large scale data to carry out high-quality conversations.
Encoder-Decoder [74]	A framework for constructing a seq2seq model which respectively takes two pretrained models as its encoder and decoder. In this experiment, we examine BERT2BERT, a model using a BERT-based [61] encoder and a BERT-based decoder.
PEGASUS [64]	A transformer-based seq2seq model for the abstractive summarization task which is pretrained using extracted gap-sentences on large scale datasets.
ProphetNet [63]	A self-supervised model with future n-gram prediction and n-stream self-attention mechanisms that is pretrained on large scale text corpora for both question generation and text summarization tasks.
T5 [65]	A large-scale pretrained transformer-based model which utilizes transfer learning techniques to convert text-related language tasks into a text-to-text format.

**Table 2:** The performance of IvCDS and baselines on three tasks, where P = precision, R = recall, F1 = F1 score, the models are sorted by the F1 score on the NLU task, and a highlighted score indicates that its corresponding model outperforms other models according to the evaluation metric.

Model	NLU			POL			NLG			
	P	R	F1	P	R	F1	BLEU-4	ROUGE-L	METEOR	GLEU
IvCDS	**92.87**	**91.71**	**92.29**	**87.23**	86.98	**87.10**	**28.19**	**49.29**	47.88	**27.80**
ProphetNet	88.65	87.86	88.25	64.18	61.51	62.81	11.23	30.29	33.63	12.69
BART-large	76.59	86.85	81.40	66.26	**89.26**	76.06	20.37	40.86	**49.75**	22.50
BART-base	68.18	89.38	77.35	46.83	88.01	61.14	19.53	40.04	49.45	21.77
Pegasus	41.33	88.62	56.37	72.31	84.33	77.86	6.39	26.53	37.97	7.77
T5-large	39.19	67.13	49.49	37.09	41.97	39.38	7.80	26.55	39.35	9.39
BigBird	48.37	43.24	45.66	81.92	72.73	77.05	20.16	37.13	39.60	20.01
Blenderbot	29.97	76.86	43.12	32.62	58.80	41.96	2.98	13.52	27.02	3.62
BERT2BERT	19.41	87.23	31.76	21.28	68.38	32.46	4.45	21.32	29.80	5.35

**Table 3:** The results of ablation experiment on the performances of IvCDS on three tasks according to various components in training/inference input sequences, where *H* is dialogue history, DP is driver profile, and highlighted numbers indicate the best model on that evaluation metric.

Training		Inference		NLU			POL			NLG			
*H*	DP	*H*	DP	P	R	F1	P	R	F1	BLEU-4	ROUGE-L	METEOR	GLEU
				78.23	88.09	82.87	18.34	24.00	20.79	1.43	10.13	11.67	2.63
✓		✓		65.24	76.79	70.54	21.43	21.55	21.49	0.93	9.27	10.55	2.14
	✓		✓	61.45	83.26	70.71	27.32	39.14	32.18	1.59	13.46	15.66	2.83
✓	✓			87.30	91.47	89.33	26.48	28.28	27.35	23.48	47.41	46.91	24.14
✓	✓	✓		92.47	91.94	92.20	28.77	31.28	29.97	26.36	48.35	47.17	26.02
✓	✓		✓	88.94	91.36	90.13	73.47	79.30	76.27	27.82	48.67	**48.43**	27.41
✓	✓	✓	✓	**92.87**	**91.71**	**92.29**	**87.23**	**86.98**	**87.10**	**28.19**	**49.29**	47.88	**27.80**

## Data Availability

Publicly available datasets were analyzed in this study. This data can be found here: https://github.com/TianboJi/ivcds (accessed on 13 October 2022).
